# What can we learn from COVID-19 vaccine R&D in China? A discussion from a public policy perspective

**DOI:** 10.1093/jtm/taab026

**Published:** 2021-02-25

**Authors:** Yinglian Hu, Simiao Chen

**Affiliations:** Chinese Academy of Governance, Beijing, China; Heidelberg Institute of Global Health (HIGH), Faculty of Medicine and University Hospital, Heidelberg University, Heidelberg, Germany; Chinese Academy of Medical Sciences and Peking Union Medical College, Beijing, China

## Abstract

Given China’s relatively weak innovative and regulatory capacity compared with developed countries, China’s progress on COVID-19 vaccines is especially impressive. We summarize three key lessons from China’s experience with COVID-19 vaccine R&D: (i) set strategic vaccine R&D goals and achieve broad consensus; (ii) strengthen coordination across government agencies and (iii) adopt the state-driven collaborative model.

Since the beginning of the coronavirus disease 2019 (COVID-19) pandemic, China has made significant progress on vaccine research and development (R&D). On 16 March 2020, China’s CanSino and Academy of Military Medical Sciences launched the world’s first COVID-19 vaccine clinical trial in Wuhan, which subsequently yielded the first human trial data on 20 May.^1^ As of 15 January 2021, out of 22 total vaccine candidates in phase 3 (or combined phase 2/3) clinical trials, 6 originated in China.^3^ And following release of interim phase 3 trial data indicating 79.34% efficacy, the National Medical Products Administration (NMPA) conditionally approved a COVID-19 vaccine developed by the China National Biotec Group (CNBG) for widespread use on 30 December 2020.^4^

However, compared with developed countries such as the USA and the UK, China generally lags behind in non-COVID-19 vaccine R&D, production and regulatory capacity. For example, in 2018, none of the top 15 vaccines in terms of global revenue was from China.^5^ Additionally, the World Health Organization’s (WHO) current framework for assessing regulatory capacity suggests that China’s national regulatory authority still needs to be improved. Given China’s relatively weak innovative and regulatory capacity compared with developed countries, China’s progress on COVID-19 vaccines is especially impressive. In this article, we summarize three key lessons from China’s experience with COVID-19 vaccine R&D.

## Lesson 1: Set Strategic Vaccine R&D Goals and Achieve Broad Consensus

During an epidemic of a new infectious disease, solely relying on market mechanisms may slow the progress of vaccine R&D due to high levels of uncertainty. Despite having access to the new virus’s genome from the beginning of the initial COVID-19 outbreak, some international pharmaceutical companies were reluctant to engage in vaccine development due to business concerns. Generally speaking, these companies dedicate significantly greater research resources to developing lucrative treatments for chronic disease such as cancer than to countermeasures for infectious diseases, especially those that primarily affect low- and middle-income countries. Janssen and Pfizer only started to take action on a COVID-19 vaccine in late February,^6^ when large-scale infection was imminent in western countries.

Soon after containing China’s initial COVID-19 outbreak, the central government set a strategic goal to maintain no or minimal local transmission until the population became protected through immunization with safe and effective vaccines.^7^ In an effort to accelerate vaccine R&D and rollout, the Ministry of Science and Technology (MOST) of China quickly initiated the emergency response research projects, 1 day before Wuhan’s lockdown and 3 months ahead of the launch of operation warp speed (OWS) in the USA. MOST would go on to sponsor five technology roadmaps and 12 vaccine candidates, including some being developed by private companies and startups. The Chinese government’s promise to protect people’s health at all expense and leaders’ credible commitment to providing COVID-19 vaccines as a global public good have motivated developers to invest substantially in vaccine R&D.

## Lesson 2: Strengthen Coordination Across Government Agencies

Heterogeneity exists in the allocation of resources, functions and information related to vaccine R&D across government agencies and institutions. In addition, stakeholder goals may vary. For instance, MOST may call on scientists to prioritize public health utility in vaccine R&D (e.g. by developing a highly efficacious and broad-spectrum influenza vaccine), even if the resulting product carries a moderate risk of adverse effects like rash or diarrhoea; regulators, on the other hand, are likely to place greater priority on vaccine safety.

**Figure 1:**
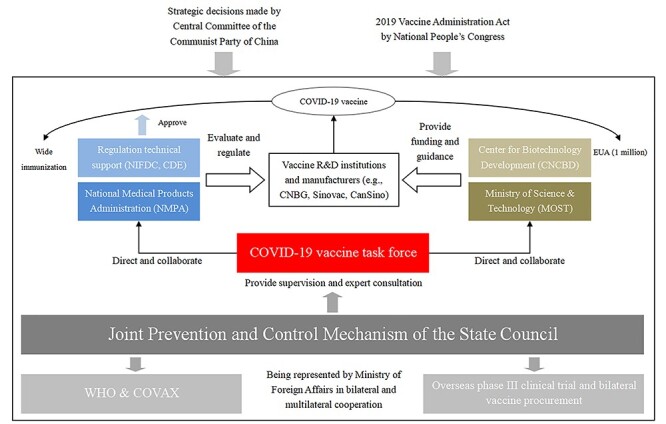
The organizational structure for COVID-19 vaccine R&D in China. *Notes:* NIFDC, National Institute for Food and Drug Control; CDE, Center for Drug Evaluation; NIFDC and CDE are directly under the NMPA, which is the regulatory authority responsible for drug approval and clinical trial registration; EUA, emergency use authorization; COVAX, COVID-19 vaccines global access; Sinovac, Sinovac Biotech Ltd—a Chinese biopharmaceutical company; CanSino, CanSino Biologics—a Chinese vaccine company

In order to coordinate policy goals across agencies and mobilize resources promptly, a COVID-19 vaccine task force was established in mid-February. The task force, which includes senior officials from NMPA, MOST and other departments, is affiliated with the Joint Prevention and Control Mechanism of the State Council and directly reports to the Vice Premier of China. Figure 1 depicts the organizational structure of COVID-19 vaccine R&D in China, including key players and their functions and relationships.

Under the task force’s direction and guidance, multiple players collaborated to maximize their joint performance. Thanks to a decades-long standing relationship between MOST and pharmaceutical companies, the Chinese government has been able to promptly identify enterprises that are capable of COVID-19 vaccine R&D during the current public health emergency. The task force has also supported the efficient allocation of experimental animal resources required for vaccine R&D across the Chinese Academy of Sciences, universities, the army and state-owned enterprises. In addition, the task force has directed NMPA to modify its procedure in accordance with the Vaccine Administration Law to streamline the inspection and review process for vaccines and dramatically accelerate market approval.

## Lesson 3: Adopt the State-Driven Collaborative Model

We have identified three categories of national vaccine R&D models: (i) the government-oriented model, which relies on political mobilization and generates knowledge mainly through academic, public sector and non-profit organizations; (ii) the market-oriented model, which relies on economic instruments (e.g. monetary incentives) and generates knowledge mainly through private developers and (iii) the state-driven collaborative model, which integrates political mobilization and economic instruments and generates knowledge through both the public and private sectors.

These three types of COVID-19 vaccine R&D models were used in different countries. For example, Russia adopted the government-oriented model, with the state-run Gamaleya Institute acting as a major developer of the Sputnik-V vaccine. Political mobilization tools, such as Russia’s Sovereign Wealth Fund, were also widely applied in the process. In contrast, the USA adopted the market-oriented model. Although OWS was launched as a public–private partnership and various federal government agencies engaged in the plan, economic instruments such as Advanced Purchase Commitments have been used widely to stimulate market competition with little direct interference from the US government. Both Pfizer and Moderna contracted with OWS to supply millions of COVID-19 vaccine doses long before they were granted an emergency use authorization or licensure by the US Food and Drug Administration.

In contrast to the countries mentioned above, China adopted the state-driven collaborative model, which leverages both government’s role and market mechanisms and has three advantages. First, it can address market failures during a public health emergency and incentivize companies to act immediately by mitigating potential risks and bolstering profits through huge public investment. Second, it can strategically concentrate resources to support a particular policy goal. Third, by integrating information, it can diminish information asymmetry and thus incentivize collaboration across all sectors.

The state-driven collaborative approach has played a role throughout the COVID-19 vaccine R&D process in China. For instance, the Beijing municipal government unconditionally funded Sinovac’s acquisition of a 69 000-square-meter vaccine manufacturing plant and related facilities, helping ensure that it can produce 300 million COVID-19 vaccine doses annually. The central government also collaborated with Chinese vaccine companies to facilitate international phase 3 clinical trials (including trials in Brazil, Turkey and Indonesia) in accordance with international rules and regulations.

## Conclusion

The COVID-19 vaccine R&D process in China reflects the cultural value of respecting life at all expense and illustrates the resilience and flexibility of the country’s governance system and market mechanisms for tackling an epidemic. In particular, China’s reliance on a state-driven collaborative model of innovation has helped accelerate the development of safe and effective vaccines. With CNBG’s inactivated vaccine recently receiving conditional approval from NMPA, we expect more high-quality and affordable COVID-19 vaccines will be delivered as global public goods in the near future.

## Authors’ Contributors

YH and SC conceived the article, conducted literature search, drew the figure and wrote the article.

## Funding

YH was supported by National Social Science Fund of China (Project 20ZDA042). SC was supported by the Alexander von Humboldt Foundation. This article was also supported by funding from the Bill & Melinda Gates Foundation (Project INV-006261), and the Sino-German Center for Research Promotion (Project C-0048), which is funded by the German Research Foundation (DFG) and the National Natural Science Foundation of China (NSFC).

## Conflict of Interest

All authors declare no competing interests.
